# Biochar Innovations for Organic Pollutant Remediation in Contaminated Soils

**DOI:** 10.3390/molecules31030432

**Published:** 2026-01-27

**Authors:** Pengfei Li, Ying Liu, Yangyang Sun, Congyu Zhang

**Affiliations:** 1 College of Geographical Science, Harbin Normal University, Harbin 150025, China; lpf@hrbnu.edu.cn (P.L.);; 2 School of Resources and Environment, Northeast Agricultural University, Harbin 150030, China

**Keywords:** biochar, organic pollutants, soil remediation, sorption and interaction processes, environmental sustainability

## Abstract

Soil contamination by organic pollutants such as polycyclic aromatic hydrocarbons (PAHs), pesticides, pharmaceuticals, and petroleum hydrocarbons has emerged as a global environmental concern due to their persistence, bioaccumulation, and potential health risks. Biochar, a carbon-rich material derived from the pyrolysis of biomass, has attracted increasing attention as an environmentally friendly and cost-effective amendment for remediating contaminated soils. This review systematically summarizes recent advances in the application of biochar for the remediation of organic pollutants in soils to guide the development of more effective biochar-based strategies for sustainable soil remediation. The physicochemical properties of biochar influencing pollutant interactions are discussed, including surface area, pore structure, functional groups, and aromaticity. Mechanisms such as adsorption, sequestration, microbial interaction enhancement, and catalytic degradation are elucidated. Moreover, this review highlights the influence of feedstock types, pyrolysis conditions, biochar modification strategies, and environmental factors on biochar performance. The analysis reveals that biochar performance is strongly dependent on feedstock selection, pyrolysis conditions, and post-modification strategies, which jointly determine pollutant immobilization efficiency and long-term stability. Current challenges, such as long-term stability, pollutant desorption, and ecological impacts, are critically examined. Finally, future perspectives on the design of engineered biochar and its integration with other remediation technologies are proposed. Rationally engineered biochar, particularly when integrated with biological or physicochemical remediation technologies, demonstrates strong potential for efficient and sustainable soil remediation.

## 1. Introduction

Soil contamination by organic pollutants has become a critical global environmental concern driven by rapid industrialization, intensive agricultural practices, and accelerated urban expansion [1]. A wide spectrum of organic contaminants such as polycyclic aromatic hydrocarbons (PAHs), polychlorinated biphenyls (PCBs), pesticides, antibiotics, and emerging persistent compounds including perfluoroalkyl and polyfluoroalkyl substances (PFASs) is continuously released into terrestrial ecosystems through industrial discharge, agricultural runoff, wastewater irrigation, and atmospheric deposition [2,3]. These pollutants commonly exhibit strong persistence, hydrophobicity, bioaccumulation potential, and acute or chronic toxicity, which present serious threats to soil ecological functioning, food security, and human health. Consequently, the development of sustainable, cost-effective, and environmentally compatible remediation strategies is urgently required [4].

Generally, remediation strategies for organic pollutants in contaminated soils can be broadly classified into physical, chemical, and biological approaches, as well as their integrated applications [5]. Physical remediation aims to remove or isolate contaminants from the soil matrix, with representative techniques including soil excavation and replacement, thermal desorption, solidification and stabilization. These methods are typically effective and rapid but are often associated with substantial site disturbance and high implementation costs. Chemical remediation achieves contaminant removal or risk reduction through oxidation–reduction reactions, chemical degradation, or adsorption-based immobilization, such as chemical oxidation, reductive dechlorination, and the amendment of sorptive materials [6]. Although conventional remediation approaches including soil washing, thermal treatment, and chemical oxidation have been widely utilized, their field-scale application is frequently limited by high operational cost, potential secondary pollution, and poor adaptability to complex soil matrices. Bioremediation relies on the degradation, transformation, or uptake of organic contaminants by microorganisms or plants and is generally considered environmentally benign and cost-effective. However, its performance is often constrained by site-specific conditions and characterized by relatively long remediation timeframes [7]. Biochar is a carbon-rich porous material produced through the pyrolysis of biomass under limited oxygen conditions, which has attracted extensive attention as a promising soil amendment for the remediation of organic contaminants [8,9,10]. Benefiting from its large specific surface area, tunable porosity, diverse surface functional groups, and strong affinity for hydrophobic compounds, biochar demonstrates significant capability to immobilize and transform organic pollutants in contaminated soils [11].

In recent years, research on biochar in the remediation of organic polluted soil has been continuously intensified, and significant progress has been made. The basic research systems have revealed the adsorption mechanism, aging behavior, and interactions between biochar and soil organic matter and microbial communities of organic pollutants. In addition, engineering methods such as surface modification, activation treatment, and composite material construction have significantly improved the remediation effect of biochar on difficult to degrade or strongly polar organic pollutants. Meanwhile, field and mesoscale experiments have gradually evaluated the long-term stability, potential ecological risks, carbon sequestration effects, and synergistic benefits of the soil fertility enhancement of biochar remediation. At this stage, this indicates that pollutant removal involves not only conventional adsorption and partitioning processes but also more sophisticated interaction pathways, including electron interactions, hydrogen bonding, electrostatic attraction, the catalytic activation of oxidants, electron transfer reactions, and microbial mediated degradation [12]. In addition, engineered biochars such as metal-impregnated, magnetized, heteroatom-modified, and composite biochars exhibit markedly enhanced reactivity, selectivity, and structural stability, which substantially improves repairing performance in persistent and emerging contaminants [13]. The main purpose of this review is to provide a focused and in-depth overview of the latest developments in the biochar remediation of organic pollutant-contaminated soil. At the same time, it should be noted that biochar has also been widely used for the remediation of soil contaminated with inorganic pollutants, especially heavy metals [14].

In the process of organic pollution soil remediation, the mechanism of action of biochar is evident in a number of aspects, including adsorption, fixation, chemical transformation, and biological synergy. Biochar has been shown to be an effective adsorbent for a variety of organic pollutants. This is due to its high specific surface area, developed pore structure, and abundant surface functional groups. The mechanisms by which biochar adsorb pollutants include hydrophobic distribution, pore filling, and π–π interactions. This ability to adsorb pollutants effectively reduces the bioavailability of the pollutants and minimizes their environmental migration risk in soil [15]. On this basis, modified or functionalized biochar can also be used as a reaction medium or catalytic carrier to participate in the redox conversion process of organic pollutants, significantly improving the remediation efficiency of recalcitrant organic pollutants. Furthermore, the utilization of biochar has been demonstrated to enhance the physical and chemical characteristics of soil, in addition to fostering the growth of microbial communities. Through the modulation of soil pH, nutrient supply, and redox conditions, it fosters the proliferation and metabolic activity of degradation-functional microorganisms, thereby facilitating a synergistic biodegradation of organic pollutants. The potential of biochar in the remediation of organic polluted soils, including polycyclic aromatic hydrocarbons, pesticides, petroleum hydrocarbons, and antibiotics, has been demonstrated by the synergy of the multiple mechanisms mentioned above. Concurrently, it has the capacity to enhance soil structure, augment fertility, and fortify carbon sequestration functions, thereby achieving a symbiotic relationship between pollution control and ecological remediation [16].

Despite rapid progress, several knowledge gaps remain unresolved. There are still many challenges in long-term environmental behavior prediction, large-scale preparation and quality consistency control, as well as ecological safety assessment under different site conditions [17]. The efficiency of biochar varies significantly depending on biomass feedstock, pyrolysis operating conditions, and soil physicochemical properties. Environmental concerns such as the potential release of inherent contaminants, long-term stability and aging effects, regeneration and reuse feasibility, and overall economic viability must be more thoroughly evaluated. Therefore, a comprehensive review is needed to synthesize the latest advances, clarify removal mechanisms, and identify priorities for future development that bridge laboratory-scale studies and real-world field applications. To this end, this review attempts to review some of the important research progress in biochar technology within the field of organic-matter-contaminated soil remediation in recent years, and discuss and summarize the key technologies related to biochar remediation. The present review provides a focused and integrative assessment of recent advances in biochar-based remediation of organic-contaminated soils. Distinct from previous reviews, this work systematically links biochar physicochemical properties with pollutant removal pathways by jointly analyzing adsorption, catalytic transformation, and biochar–microbe interaction mechanisms. In addition, recent progress in modified and composite biochar materials is critically evaluated, and remediation performance is comparatively discussed across major classes of organic pollutants. Particular emphasis is placed on identifying limitations, unresolved knowledge gaps, and key challenges in scaling up biochar applications from laboratory studies to field-scale implementation. Overall, this review aims to deliver both mechanistic insights and practical guidance to support the development and deployment of effective and sustainable biochar-based soil remediation strategies.

The rest of this review is organized as follows. First, the fundamentals and physicochemical properties of biochar for organic pollutant remediation are introduced. Subsequently, the typical biochar in adsorption and immobilization mechanisms is reviewed. The driving mechanisms of biochar–microbe interaction in soil is analyzed. The key application issues of biochar in soil remediation are discussed, and then a summary of the corresponding solutions is provided. Furthermore, the priorities and trends of biochar for the soil remediation of organic pollutants are indicated. Finally, a conclusion is given.

## 2. Methodology

**Figure 1:**
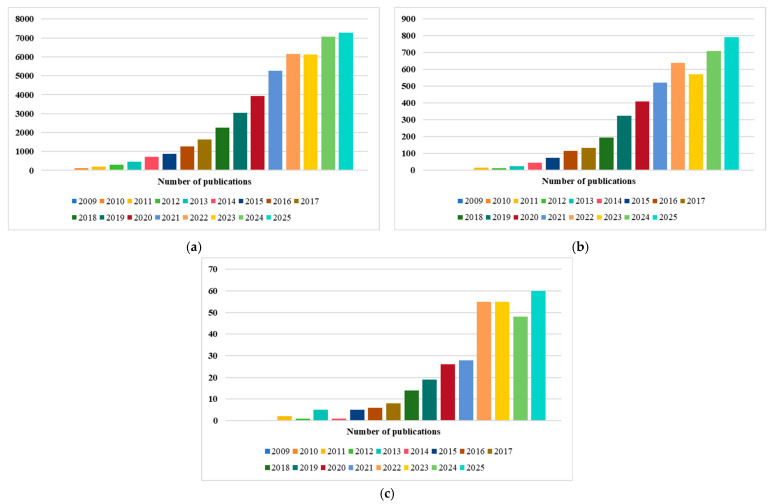
Publication trends during the study period (2009–2025). (**a**) The result of searching for the keyword “Biochar”; (**b**) The results of searching for keywords “Biochar” and “soil remediation”; (**c**) The results of searching for keywords “Biochar”, “soil remediation” and “organic pollutants”.

A search of the scientific literature was conducted to explore available and relevant reviews and articles on biochar in soil remediation for organic pollutants. According to “Web of Science Core Collection” database, the result of searching for the keyword “Biochar” is shown in Figure 1a. The results of searching for keywords “Biochar” and “soil remediation” are shown in Figure 1b, and a total of 4631 papers published over the past 17 years (2009–2025) are available. The results of searching for keywords “Biochar”, “soil remediation” and “organic pollutants” are shown in Figure 1c, and it reveals an overall upward trend in research on organic pollutants in soil, with a slight decline observed in 2024. These trends reflect a growing interest in soil remediation of organic pollutants over the past ten years and the accumulation of substantial research findings.

## 3. Biochar Fundamentals and Physicochemical Properties

The basic characteristics of biochar are largely influenced by factors such as material type, conversion temperature, heating rate, and residence time, which determine the carbon structure characteristics, mineral composition, and surface chemical properties of biochar [18]. These inherent characteristics constitute the material basis for the long-term stable existence of biochar in soil environment and its interaction with organic pollutants. Below, the preparation of biochar is reviewed from the perspectives of feedstock sources and production methods, followed by a discussion of its physicochemical properties and pollutant removal performance.

### 3.1. Feedstock Sources

Common biochar raw materials can be roughly divided into woody biomass, agricultural residues, livestock manure, and other organic waste. The applicability of biochar prepared from different types of raw materials in organic pollutant remediation varies significantly. Wood based biomass, such as sawdust, tree branches, and forestry waste, typically has high lignin content and are prone to form biochar with high aromatization and strong structural stability during pyrolysis. This type of biochar often has a large specific surface area and well-developed microporous structure, exhibiting strong adsorption capacity for hydrophobic organic pollutants such as polycyclic aromatic hydrocarbons and petroleum hydrocarbons. It is suitable for remediation scenarios aimed at pollutant fixation and long-term stability.

Agricultural residues come from a wide range of sources and have cost advantages, making it the most common biochar raw material in current research and application. Among them, rice husks, straw (wheat straw, corn straw, and cotton straw), corn cobs, and grass are used for the preparation of biochar [19]. Biochar prepared from such raw materials usually retains more oxygen-containing functional groups and mesoporous structures, with strong surface chemical activity, which is conducive to adsorbing polar or semi polar organic pollutants. In addition, its ash content and alkaline characteristics can regulate the soil physicochemical environment to a certain extent, thereby enhancing the synergistic effect of bioremediation. Nutritious organic waste, represented by livestock manure and sludge, usually contains a high proportion of inorganic mineral components and metal elements, and the biochar prepared often exhibits high ash content and alkalinity [20]. In addition to adsorption, this type of biochar may also exhibit certain catalytic or redox activity in organic pollutant remediation, making it suitable for composite remediation systems combined with chemical oxidation or bio enhanced remediation technologies. However, its potential heavy metal risks and environmental safety still require attention in practical applications.

The source of raw materials affects the carbon skeleton structure, pore characteristics, surface functional group composition, and mineral phase characteristics of biochar, thereby determining its mode of action and application potential in organic pollutant remediation. Raw materials with high lignin content, such as wood and fruit shells, tend to produce biochar with more developed pore structure and higher carbon stability. In contrast, raw materials with high cellulose and ash content, such as straw and livestock manure, may produce biochar with richer surface functional groups but higher ash content. Xiao et al. revealed the conversion patterns of silicon and carbon at different temperatures for rice straw, which affects the stability and surface properties of biochar [21]. Zhuang et al. synthesized rice husks, corn stalks, and pine sawdust biochar at different pyrolysis temperatures and subjected them to ball milling treatment. The results indicate that wooden biochar has a high adsorption capacity for volatile organic compounds, and the adsorption rates of all ball milled biochar are higher than those of the original biochar, which can serve as potential adsorption materials for volatile organic compound treatment [22]. Combining nanomaterials, Zhuo et al. prepared a composite material of nano-zero-valent iron/biochar, achieving a synergistic effect of adsorption and reduction dechlorination of chlorinated hydrocarbons in soil [23].

### 3.2. Production Methods

The preparation method of biochar is an important factor determining its structural characteristics, surface chemical properties, and environmental functions. There are significant differences in reaction temperature, pressure conditions, residence time, and medium environment among different thermal conversion pathways, resulting in significant differences in carbon structure stability, pore development degree, and functional group composition of the prepared biochar. Biochar is typically produced through thermochemical conversion of biomass under oxygen-limited conditions. The main production technologies include pyrolysis, hydrothermal carbonization, torrefaction, and emerging processes such as flash carbonization, whereas gasification primarily targets syngas production and generates biochar as a secondary by-product [24,25,26].

Pyrolysis is usually carried out under anaerobic or oxygen limited conditions within the range of 300–700 °C, and is the mainstream process for preparing biochar. Biochar prepared by low-temperature pyrolysis often retains more oxygen-containing functional groups and has strong surface chemical activity, which is conducive to the adsorption and interaction of polar organic pollutants. High temperature pyrolysis significantly improves the aromatization degree and structural stability of biochar, promotes the development of microporous structure, and enhances its adsorption capacity for hydrophobic organic pollutants. Considering different operating parameters, the pyrolysis process is categorized into fast pyrolysis, slow pyrolysis, microwave-assisted pyrolysis, and catalytic pyrolysis. According to Chen et al. [27], the adsorption capacity of pyrene by rice husk biochar pyrolyzed at 700 °C is 3.5 times that of biochar prepared at 400 °C, mainly attributed to the higher specific surface area and graphitization degree of the former. Das et al. evaluated the effects of raw materials and pyrolysis temperature on 36 compositional characteristics of biochar. They found that as the pyrolysis temperature increased from 400 °C to 600 °C, the total carbon content of biochar studied increased by 10.14%. The type of raw material had a more significant impact on the properties of biochar than pyrolysis temperature [28]. With the increase in pyrolysis temperature, the aromatization degree and carbonization level of biochar are significantly enhanced, and its structural stability continues to improve, while the content of oxygen-containing functional groups and volatile components decreases accordingly.

Gasification is usually carried out at higher temperatures (>700 °C) and with limited participation of oxygen or water vapor, with the main goal of energy recovery. However, the resulting by-product biochar, also known as gasification charcoal, typically has higher ash content and alkaline characteristics. The pore structure of this type of biochar is relatively irregular, with significant differences in specific surface area. However, its surface mineral composition and alkaline characteristics can provide additional catalytic or environmental regulation functions in certain organic pollutant remediation systems. The higher equivalency plays a key role in gasification process production and carbon sequestration. Yao et al. found that there is an inverse relationship between the equivalence ratio, the carbon content of the product, and the yield of the gasification process [29]. Regarding the co-production of biochar and syngas through gasification, Zhou studied two by-products of pine wood particle gasification, namely biochar and syngas [30]. The results indicate that gasification increases the pore structure of biomass charcoal, and by controlling gasification parameters such as temperature, steam volume, and air equivalence ratio, the product distribution can be effectively controlled.

**Figure 2:**
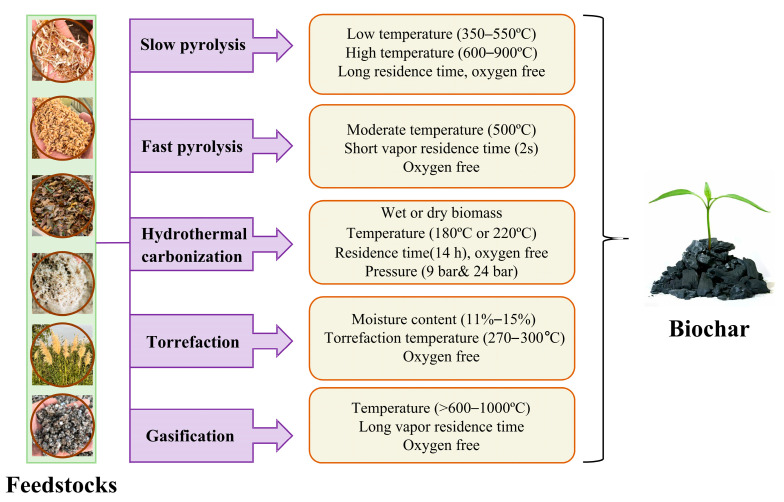
Processes to biochar preparation through different feedstocks (modified from [31]).

Hydrothermal carbonization is a conversion process completed in an aqueous environment under relatively low temperature (180–250 °C) and autogenous pressure conditions, particularly suitable for high moisture biomass raw materials. Hydrothermal carbon usually has a lower degree of aromatization and more oxygen-containing functional groups, with a relatively limited specific surface area [25]. The surface chemical activity is high, and it has certain potential in adsorbing polar organic pollutants and regulating soil physicochemical properties. However, its structural stability and long-term environmental behavior still need further evaluation. Based on each process discussed above, the processes to biochar preparation from different feedstocks are presented in Figure 2.

### 3.3. Resulting Physicochemical Characteristics

Approximately 70–75 wt% of biochar contains carbon, with the remaining portion composed of hydrogen, oxygen, and nitrogen components [32]. The physicochemical characteristics of biochar are jointly governed by feedstock sources and preparation processes, which determine its structure, surface chemistry, and environmental reactivity. Consequently, biochars produced via different raw materials and conversion pathways show pronounced differences in pore architecture, carbon structural order, functional group composition, and mineral phases, leading to distinct remediation performances. Table 1 highlights the physicochemical characteristics of biochars.

In terms of structural characteristics, biochar typically exhibits a multi–level pore system composed of micropores, mesopores, and macropores, with a significant increase in specific surface area and pore volume with increasing pyrolysis temperature [33]. The highly aromatic carbon structure formed under high temperature conditions has stronger chemical inertness and long–term stability, which is conducive to the adsorption and fixation of hydrophobic organic pollutants, while low–temperature biochar relies more on surface chemical interactions to participate in pollutant interactions due to limited pore development [34].

The surface chemical properties are a key factor affecting the interaction between biochar and organic pollutants. The surface of biochar is rich in oxygen–containing functional groups such as hydroxyl, carboxyl, carbonyl, and phenolic hydroxyl groups, and their abundance and reactivity are significantly affected by thermal conversion conditions [35]. Biochar prepared at low temperatures typically retains more multipole functional groups, which facilitate the adsorption of polar organic pollutants through hydrogen bonding and electrostatic interactions. The surface of high–temperature biochar is mainly composed of aromatic structures, which are more prone to π–π electron interactions and have stronger affinity for non–polar or weakly polar organic compounds.

In addition to carbon structure and functional groups, the mineral phase and ash composition in biochar also have a significant impact on its physicochemical properties. Biochar rich in inorganic minerals usually exhibits high alkalinity, ion exchange capacity, and conductivity, which can not only regulate soil acid–base conditions, but also give biochar certain catalytic or redox activity, thereby affecting the transformation behavior of organic pollutants [36]. The redox activity, surface charge characteristics, and hydrophobicity of biochar jointly determine its adsorption kinetics, stability, and long–term environmental behavior in complex soil environments.

**Table 1:** Physicochemical Characteristics of biochars.

Property Category	References	Specific Parameter	Typical Characteristics	Functional Effects	Major Pollutant/Interaction Mechanisms
Physical properties	[25,33]	Specific surface area (SSA)	Increases markedly with increasing pyrolysis temperature	Provides abundant adsorption sites and enhances sorption capacity for organic pollutants	PAHs, petroleum hydrocarbons, and mixed organic contaminants/ Pore filling, physical adsorption, microbial colonization, and enhanced biodegradation
	[34]	Pore volume and pore size distribution	Dominated by micropores and mesopores	Controls diffusion, retention, and accessibility of pollutant molecules	
	[5]	Bulk density	Generally low	Improves soil aeration and structural properties	
	[36]	Particle size distribution	Highly dependent on feedstock and post–treatment	Affects mixing uniformity and contact efficiency with soil	
Chemical properties	[37]	pH	Typically neutral to strongly alkaline	Alters soil acidity and pollutant speciation	Aromatic pesticides, antibiotics, halogenated organics, and dyes/ π–π electron donor–acceptor interactions, and catalytic transformation
	[38]	Ash content	Increases with pyrolysis severity	Supplies mineral components that facilitate precipitation and complexation	
	[39,40]	Elemental composition (C, H, O, N)	High carbon content and low H/C ratio	Indicates aromaticity and chemical stability	
	[41]	Atomic ratios (H/C, O/C)	Low H/C and decreasing O/C with temperature	Reflects carbonization degree and surface polarity	
Surface chemical properties	[42]	Surface functional groups	Presence of hydroxyl, carboxyl, and carbonyl groups	Promote hydrogen bonding, electrostatic interaction, and surface complexation	Polar or ionizable organics and redox–sensitive pollutants/ Hydrogen bonding, electrostatic interactions, electron shuttling, and mediated biodegradation
	[21,43]	Surface charge	Strongly pH–dependent	Governs electrostatic attraction or repulsion of ionizable pollutants	
	[18]	Aromaticity	Higher aromaticity at elevated pyrolysis temperatures	Enhances affinity for hydrophobic organic contaminants	

From a mechanistic perspective, the remediation performance of biochar is governed by a combination of physicochemical adsorption, catalytic transformation, and biochar–microbe interactions rather than isolated material properties. High aromaticity and condensed carbon structures primarily control the affinity toward hydrophobic organic pollutants through π–π electron donor–acceptor interactions, whereas surface functional groups, such as hydroxyl, carboxyl, and nitrogen–containing moieties, regulate polarity–dependent interactions including hydrogen bonding and electrostatic attraction. Pore structure and surface area further determine mass transfer efficiency and accessibility of active sites, thereby influencing both adsorption capacity and kinetic behavior. In addition, modified and composite biochars introduce catalytic or redox–active sites that facilitate abiotic transformation of organic contaminants, while biochar–mediated changes in soil microhabitats enhance microbial colonization and enzymatic activity, promoting coupled biotic degradation pathways. These mechanisms act synergistically and are strongly modulated by environmental conditions, including soil pH, moisture, and coexisting organic matter.

## 4. Adsorption and Immobilization Mechanism

**Figure 3:**
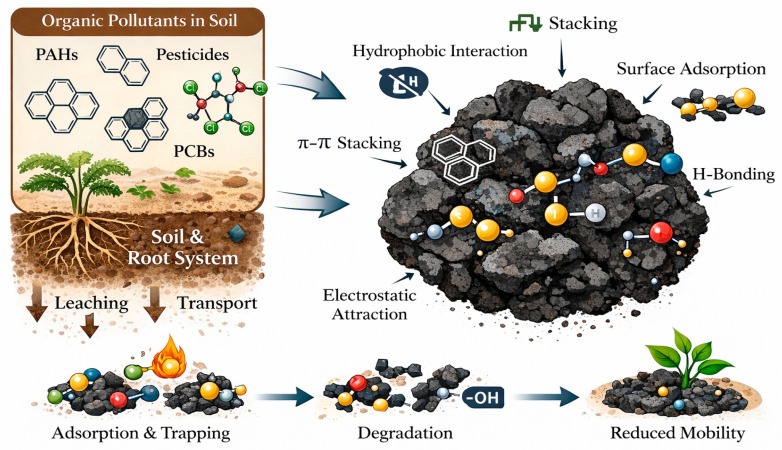
Schematic diagram of biochar adsorption mechanism for organic pollutants in soil.

Biochar is characterized by a high specific surface area, strong aromaticity, abundant polar functional groups, and excellent adsorption capacity, which collectively contribute to its effective removal of a wide range of organic pollutants. However, the adsorption performance of biochar is strongly dependent on the physicochemical properties and types of target pollutants, highlighting the necessity of systematically investigating biochar–pollutant interactions for specific contaminants [44]. Accordingly, the adsorption mechanisms of organic pollutants in biochar–amended soils are discussed from three main perspectives: hydrophobic and π–π interactions, interfacial chemical interactions, as well as pore confinement and synergistic effects. Figure 3 illustrates the adsorption mechanism of biochar on organic pollutants in soil.

Adsorption has been widely recognized as an effective and versatile strategy for the removal of organic pollutants from contaminated environments. Owing to the substantial variability in surface chemistry, pore structure, and functional group composition among biochars derived from different feedstocks and preparation conditions, the adsorption mechanisms of organic pollutants can differ markedly across biochar types. A systematic analysis of these mechanisms is therefore crucial for elucidating the key factors governing biochar–pollutant interactions and for guiding the rational design and application of biochar–based remediation strategies. In general, the adsorption of organic pollutants onto biochar involves multiple mechanisms, including electrostatic interactions, hydrophobic interactions, pore filling, hydrogen bonding, and π–π electron donor–acceptor interactions.

### 4.1. Hydrophobic and π–π Interactions

Hydrophobic partitioning and π–π electron donor–acceptor interactions constitute the dominant mechanisms governing the adsorption of nonpolar and weakly polar organic pollutants in biochar–amended soils. Owing to its high aromaticity and carbon content, biochar provides hydrophobic domains that favor the partitioning of organic contaminants from the aqueous phase onto solid surfaces, particularly for compounds with high octanol–water partition coefficients and low water solubility, such as polycyclic aromatic hydrocar, chlorinated hydrocarbons, and certain pesticides [38]. The π–π interaction is an important chemical mechanism for the binding of aromatic organic pollutants with biochar. High temperature pyrolysis biochar usually has a high degree of aromatization and conjugated structure, and its surface is rich in π–electron systems, which can undergo π–π stacking or electron donor acceptor interactions with organic pollutants containing aromatic rings. This non covalent interaction not only enhances the adsorption strength, but also improves the fixation stability of pollutants on the surface of biochar, which is of great significance in the remediation of polycyclic aromatic hydrocarbons, dyes, and some pesticide pollution. The combined effects of hydrophobic partitioning and π–π interactions not only increase adsorption capacity but also promote stronger binding and reduced desorption, thereby decreasing pollutant mobility and bioavailability in soil environments.

Fan et al. concluded through model calculations and experimental verification that the adsorption of tetracycline by magnetic straw biochar is dominated by pore filling and π–π interactions, while hydrogen bonding and complexation also contribute [45]. To effectively utilize a large amount of fruit shell waste, Zhang et al. converted it into biochar to remove high levels of bromophosphorus. The research results indicate that the adsorption mechanism of propiconazole on fruit shell biochar is mainly through π–π and hydrophobic interactions, and the prepared biochar exhibits strong adsorption capacity for aromatic organophosphorus pesticides [46]. Zhong et al. have prepared a novel biochar that combines mineral composites. The results showed that hydrophobic interactions controlled the adsorption of TPhP and TPPO on composite biochar, and π–π interactions, hydrogen bonding also participated in the adsorption process [47].

### 4.2. Interfacial Chemical Interactions

**Figure 4:**
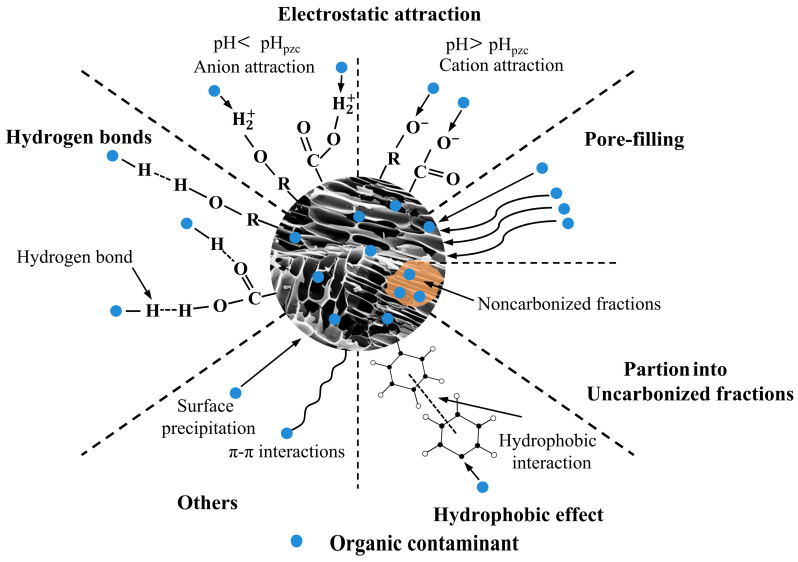
The mechanism of biochar removal of organic pollutants from soil.

The oxygen–containing functional groups and mineral components on the surface of biochar jointly regulate its adsorption behavior towards polar and ionizable organic pollutants. Functional groups such as hydroxyl, carboxyl, and carbonyl can bind to pollutants through hydrogen bonding, electrostatic interactions, and coordination, while ash and mineral phases may enhance adsorption efficiency through surface complexation, ion exchange, and local pH adjustment. Biochar prepared at low temperatures or modified typically has a higher abundance of functional groups, exhibiting superior performance in the fixation of pesticides, antibiotics, and emerging organic pollutants. The mechanisms employed for organic pollutants removal in soil by biochar are illustrated in Figure 4.

The soil remediation ability of a modified manure based biochar was investigated, and the results showed that various oxygen–containing surface functional groups on the biochar surface, such as phenols, carboxyl groups, and carbonyl groups, may act as proton donors for pollutant adsorption [42]. Dong et al. explored the adsorption mechanism and ecological benefits of hydrogen peroxide modified biochar in reducing temotrigine pollution in soil environment. Density functional theory calculations and non–covalent interaction analysis in the study showed that hydrogen bonding and π–π stacking were the main adsorption mechanisms [48]. In addition, oxidation modification can introduce rich oxygen–containing functional groups, including hydroxyl, carboxyl, and carbonyl groups, greatly improving the adsorption capacity and surface reactivity of biochar. Zhong et al. attempted to prepare biochar by low–temperature (280 °C) phosphoric acid assisted pyrolysis of cotton stalks, which has a large specific surface area, rich oxygen functional groups, and phosphorus and nitrogen nutrients. The study explored adsorption mechanisms including pore filling, hydrogen bonding, weak electrostatic effects, and π–π interactions, and the results showed that the prepared biochar can alleviate the phytotoxicity of paclobutrazol (PBZ) to mung bean seedlings, ensuring the normal growth of roots and plants [49].

### 4.3. Pore Confinement and Synergistic Effects

Pore filling and hydrophobic distribution are the fundamental mechanisms for the adsorption of organic pollutants by biochar. Biochar forms a multi–level pore structure dominated by micropores and mesopores during the thermal conversion process, providing a large amount of physical adsorption space for organic pollutant molecules. Hydrophobic organic pollutants tend to migrate from soil solutions to the interior of the biochar carbon skeleton and enter the pore system through hydrophobic distribution, thereby achieving stable interception. This mechanism is particularly significant for non–polar organic pollutants such as polycyclic aromatic hydrocarbons and petroleum hydrocarbons, and its adsorption capacity usually increases with the increase in biochar specific surface area and aromatization degree.

In soil environment, the adsorption of biochar ultimately manifests as effective fixation of organic pollutants and reduction in environmental risks. By inhibiting the desorption, leaching, and biological absorption processes of pollutants, biochar significantly reduces its bioavailability and mobility. In addition, the synergistic effect of multiple adsorption mechanisms and the aging behavior of biochar in soil jointly determine the long–term stability of pollutant fixation. Therefore, a systematic understanding of the adsorption and fixation mechanism of biochar is crucial for evaluating its long–term remediation effectiveness and engineering applicability.

In most cases, the interaction between biochar and soil is regulated by the high SSA, which is mainly influenced by the type of feedstock biomass and the pyrolysis conditions during the preparation process [50]. Duan et al. found that eucalyptus based biochar prepared at 800 °C has more micropores than biochar prepared at 400 °C, indicating a greater tendency to adsorb C_9_H_10_C_l2_N_2_O from soil [51]. Yang et al. utilized molecular dynamics simulations and density functional theory calculations to investigate the adsorption behavior of boron modified biochar. The results showed that the prepared biochar significantly improved its adsorption capacity by optimizing its pore structure and surface function, promoting rapid microporous filling, and strong electrostatic interactions [52]. Wang et al. employed KHCO_3_ as a pore forming agent to prepare a novel porous biochar from biological waste through pyrolysis. The results indicate that pore filling and π–π electron donor–acceptor interactions are the main mechanisms for the adsorption of organic pollutants by the novel porous biochar, while hydrogen bonding and electrostatic interactions only partially participate in the adsorption process [53].

The adsorption and immobilization of organic pollutants in biochar–amended soils result from the synergistic action of multiple physical and chemical mechanisms. The high surface area, aromatic structure, and functionalized surfaces of biochar promote hydrophobic partitioning, π–π interactions, hydrogen bonding, electrostatic attraction, and surface complexation, while pore filling further limits pollutant desorption and mobility. Collectively, these processes reduce pollutant bioavailability and govern the long–term remediation performance of biochar in contaminated soils.

## 5. Driving Mechanisms of Biochar–Microbe Interaction in Soil

**Figure 5:**
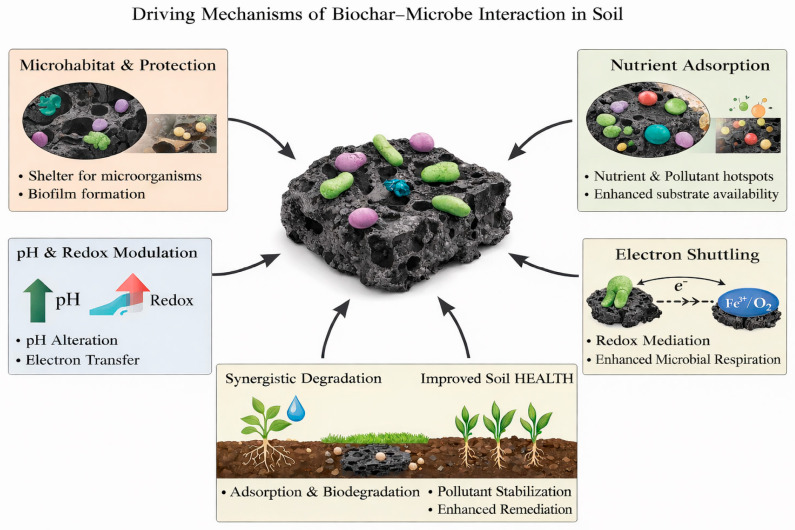
Schematic diagram of driving mechanisms of biochar–microbe interaction in soil.

The driving mechanisms of biochar–microbe interaction in soil are governed by the inter–play between biochar’s structural properties, surface chemistry, and environmental modulation effects. Pollutant immobilization is mainly controlled by the physical and physicochemical properties of biochar, such as surface adsorption, pore filling, electrostatic interactions, and reductions in contaminant mobility and bioavailability. These processes can effectively alleviate acute toxicity and improve microbial habitat conditions, yet they do not involve chemical transformation of the contaminants. In contrast, true biodegradation is governed by microbial metabolic activity, including enzymatic transformation and mineralization of pollutants into less toxic or stable end products. In biochar–microbe systems, immobilization often functions as a facilitating or preparatory step by reducing toxicity and stress, whereas biodegradation represents the ultimate remediation pathway determining long term soil functionality and ecological risk. Explicitly differentiating these processes enables more accurate interpretation of remediation mechanisms and avoids overestimating biological degradation when observed effects are primarily driven by physicochemical sequestration [54]. Figure 5 illustrates the driving mechanisms of biochar–microbe interaction in soil. In fact, the introduction of biochar into soil alters the physicochemical environment and creates new ecological niches, thereby directly and indirectly influencing microbial abundance, community composition, metabolic activity, and functional potential. These interactions are driven by a combination of structural, chemical, and ecological mechanisms that operate across multiple spatial and temporal scales. Biochar–microbe interactions play a critical role in regulating soil biogeochemical processes and determining the effectiveness of biochar–assisted remediation of contaminated soils [55].

Primary mechanisms are mainly governed by the physical and physicochemical attributes of biochar, including pore architecture, specific surface area, surface charge, and the abundance of surface functional groups, which directly control contaminant adsorption and immobilization while also shaping microbial habitat availability. These mechanisms typically prevail in soils characterized by low biological activity, limited organic carbon supply, or high contaminant loads, where rapid sorption, toxicity mitigation, and microhabitat protection are essential for short–term risk reduction. In contrast, secondary mechanisms are associated with biochar–mediated redox processes, notably electron shuttling through redox–active functional groups such as quinone and phenolic moieties, and the consequent stimulation of microbially driven redox transformations. Such mechanisms become increasingly important in biologically active soils containing redox–sensitive pollutants, including multivalent metals or reducible organic contaminants, where sufficient electron donors or acceptors and favorable moisture and pH conditions are present. Under these circumstances, biochar acts not only as a passive sorbent but also as an active redox mediator, linking soil geochemical processes with microbial metabolism and facilitating longer–term contaminant transformation and detoxification.

One of the primary driving mechanisms is the habitat and refuge effect provided by biochar’s porous structure. The well–developed micro– biochar and mesopores of biochar offer protected microhabitats that shield microorganisms from environmental stresses such as predation, desiccation, and toxic pollutants. This physical protection enhances microbial survival and colonization, particularly for pollutant–degrading bacteria, and promotes the formation of biofilms on biochar surfaces. The extent of this effect is closely related to biochar pore size distribution, surface roughness, and particle morphology [56]. Surface chemistry–mediated interactions further govern biochar–microbe relationships. Biochar surfaces are enriched with oxygen–containing functional groups that facilitate microbial attachment through electrostatic interactions, hydrogen bonding, and extracellular polymeric substances. In addition, biochar can adsorb organic pollutants and nutrients, creating localized hotspots with elevated substrate availability. This adsorption–driven concentration effect enhances microbial access to carbon sources and co–metabolites, thereby stimulating microbial activity and pollutant biodegradation under favorable conditions [57].

Biochar also influences microbial processes through modification of soil physicochemical properties, including pH, redox potential, moisture retention, and nutrient availability. Many biochars exhibit alkaline characteristics that can alleviate soil acidity and create more favorable conditions for microbial growth and enzyme activity. Furthermore, biochar improves soil aeration and water–holding capacity, which supports aerobic microbial metabolism and stabilizes microbial functioning under fluctuating moisture regimes. Another important driving mechanism is electron transfer and redox mediation. Biochar contains redox–active functional groups and persistent free radicals that can participate in electron shuttling between microorganisms and electron acceptors or donors. This property facilitates microbial respiration, enhances extracellular electron transfer, and accelerates redox–driven transformation of organic pollutants. Such mechanisms are particularly relevant in anaerobic or redox–sensitive soil environments. According to Kumar et al., the increase in temperature of the aqueous solution promotes higher adsorption, while higher pH values and dissolved organic matter and nutrients may reduce the adsorption capacity of micro nano plastics using biochar. In addition, in saturated columnar porous media, biochar can inhibit various microplastics due to electrostatic repulsion, steric hindrance, and reduced competitive adsorption of humic acid, ion strength, and cations [37]. The dissolved organic matter produced by biochar has biological reactivity, which can stimulate the microbial reduction of Fe (III) in clay minerals and may provide new insights into the application of biochar in soil element cycling [58]. Through pot experiments, Liu et al. studied the effects of biochar and no nitrogen fertilizer on soil microorganisms and microfauna. They observed that a large amount of biochar increased crop productivity, microbial activity, biomass carbon and nitrogen, and the abundance of protozoa, but decreased the abundance of bacterial, fungal, and herbivorous nematodes [59].

In addition, synergistic biochar–microbe interactions emerge from the coupling of adsorption and biodegradation processes. Biochar immobilizes organic pollutants, reducing acute toxicity to soil microorganisms, while simultaneously providing a platform for microbial colonization and enzymatic transformation. This synergy enhances pollutant stabilization and long–term remediation efficiency, highlighting the importance of considering biochar–microbe interactions as an integrated system rather than isolated processes. The addition of biochar could significantly change the abundance, diversity and community composition of soil bacteria and fungi. It tends to enrich microbial groups with the function of degrading specific pollutants, such as Sphingomonas and Rhodococcus, which degrade polycyclic aromatic hydrocarbons. Pseudomonas and Bacillus degrade pesticides. This enrichment may be due to the direct adsorption of pollutants by biochar, which reduces its biological toxicity and provides favorable living conditions and additional carbon sources. According to Xiong et al., biochar inoculated with Mycobacterium can significantly enhance the biodegradation of polycyclic aromatic hydrocarbons in contaminated soil [60]. Biochar can promote the release of plant root exudates and enhance the recruitment ability for beneficial microorganisms. According to Jin et al., biochar treatment increases the organic acid content in tomato root exudates by 30%. These substances can specifically attract disease suppressing microorganisms such as Pseudomonas, forming a synergistic network of “biochar root exudates microorganisms”, inhibiting the growth of soil borne pathogens, and accelerating the degradation of organic pollutants [61]. To solve the problem that the efficiency of microbial assisted phytoremediation in soil is not ideal, Xiang et al. integrated plant growth promoting bacteria and biochar to achieve the sustainable restoration and management of soil [62].

From a molecular–level perspective, the regulatory effects of biochar on pollutant transformation and microbial processes in soils are primarily governed by its abundant and structurally diverse redox–active functional groups and the associated electron transfer behaviors. Biochar commonly contains quinone/semiquinone moieties, phenolic hydroxyl groups, and oxygen–containing heterocycles, which can function as reversible electron donor–acceptor pairs, thereby conferring a stable redox–buffering capacity. As a result, biochar can act as an effective electron shuttle within soil systems. By facilitating extracellular electron transfer, these functional groups establish efficient electron–transfer pathways between microorganisms and terminal electron acceptors, such as organic pollutants, metal oxides, or humic substances, thereby regulating microbial respiratory pathways and energy metabolism. Moreover, electron shuttling can accelerate specific redox reactions, alter pollutant transformation pathways and kinetics, and indirectly reshape microbial metabolic fluxes and functional traits. Through this coupled interaction among functional group chemistry, electron transfer, and microbial metabolism, biochar exerts a coordinated control over soil redox conditions and pollutant fate, providing a more mechanistic and molecularly grounded framework for understanding its role in soil remediation.

It should be noted that pollutant immobilization primarily refers to the physical or chemical sequestration of contaminants through mechanisms such as adsorption, pore filling, hydrophobic partitioning, and complexation with surface functional groups, which effectively reduce pollutant mobility and bioavailability without altering their molecular structure. In contrast, true biodegradation involves the biochemical transformation or mineralization of pollutants driven by microbial metabolism, leading to the breakdown of complex organic compounds into simpler, less harmful products. While biochar can indirectly promote biodegradation by enhancing microbial habitat, nutrient availability, and redox conditions, immobilization alone does not guarantee long–term detoxification. Therefore, distinguishing these two processes is essential for accurately evaluating remediation effectiveness, environmental risks, and the sustainability of biochar–based soil remediation strategies.

While biochar generally provides favorable habitats and electron transfer pathways for microorganisms, excessive adsorption strength or the presence of inhibitory compounds may also limit microbial accessibility to pollutants or suppress microbial activity under certain conditions.

Overall, the driving mechanisms of biochar–microbe interactions in soils are inherently multifactorial, arising from the combined effects of biochar porous structure, surface chemistry, and its ability to modify the soil physicochemical environment. By providing protected microhabitats, promoting microbial attachment, regulating nutrient and pollutant availability, and mediating redox and electron transfer processes, biochar exerts a profound influence on microbial survival, community composition, metabolic activity, and functional potential. These interactions form the mechanistic basis for the synergistic coupling of pollutant adsorption and microbial biodegradation, thereby improving the stability and overall efficiency of organic contaminant remediation in soils. A comprehensive understanding of these interconnected processes is therefore critical for the rational design of biochar materials and the optimization of biochar–microbe–assisted remediation strategies across diverse soil systems.

## 6. Application of Biochar in Soil Remediation

Owing to the frequent use of pesticides, on–site discharge of organic pollutants, oil spills, and deposition of atmospheric pollutants, the degree of soil organic pollution has become increasingly severe. Considering mutagenicity or carcinogenicity, many organic pollutants pose significant hazards. Among them, a considerable portion of organic pollutants are difficult to degrade [63]. Persistent organic pollutants (POPs) accumulate in soil layers rich in organic matter and remain for several years, including PAHs, PCBs, polychlorinated dibenzo–p–dioxins, and polychlorinated dibenzofurans. According to Zhai et al., the strain was immobilized with corn straw biochar to enhance its degrading of nicosulfuron. This biochar–based immobilization markedly accelerated nicosulfuron dissipation, reducing its half–life in soil from 22.8 days to 5.1 days, while simultaneously restoring the activities of key soil enzymes, including urease, sucrase, dehydrogenase, and catalase, which are closely associated with soil pollutant degradation [64]. To promote nutrient maintenance for microbial and plant growth, crop residue biochar and fecal biochar with high pyrolysis temperature can improve soil fertility. High temperature biochar derived from wood has a stable carbon structure and a high C/N, which enhances carbon sequestration and makes it less susceptible to microbial decomposition. Considering that the relatively high levels of small molecule volatile organic compounds on low–temperature biochar can serve as microbial inhibitors, it may have advantages in controlling soil borne pathogens [54]. The newly emerging pollutants are also considered to have potential hazards to animals and humans, such as triclosan and trimethoprim, natural estrogen steroids and phthalates, and so on [65].

**Figure 6:**
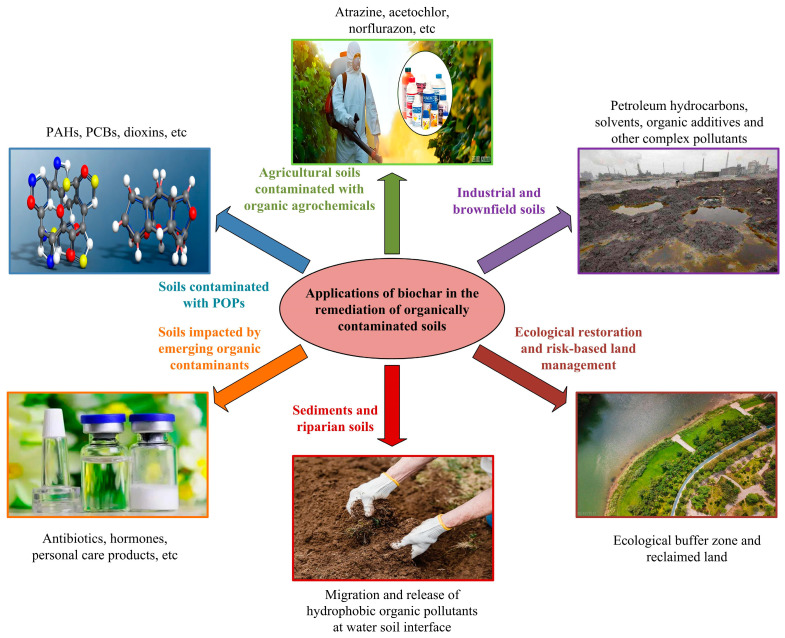
Applications of biochar in the remediation of organically contaminated soils.

The performance of biochar in organic pollutant removal is influenced by its physicochemical properties, pollutant types, and soil environmental conditions. Biochar typically exhibits high removal efficiency for hydrophobic organic pollutants, while its remediation effect on polar or ionizable organic compounds is more sensitive to their surface functional groups and modification methods. Through the coupling of various mechanisms such as adsorption fixation, catalytic conversion, and biological synergy, biochar can significantly reduce the mobility and bioavailability of organic pollutants in soil. In actual soil environments, pH, organic matter content, ion strength, and microbial activity can significantly affect the remediation effect of biochar. After the application of biochar, soil physicochemical conditions and microbial community structure are changed, which not only directly affects pollutants, but also indirectly regulates their degradation pathways and environmental fate. Therefore, the evaluation of biochar removal performance should be combined with long–term behavior and ecological risks, rather than limited to short–term removal rates. Kong et al. cultured petroleum contaminated soil using sawdust and wheat straw biochar prepared at 300 °C and 500 °C. The results indicate that biochar has the potential to promote the biodegradation of polycyclic aromatic hydrocarbons, which may be due to the properties of biochar being conducive to making the improved soil a better habitat for microorganisms [66]. According to Qian et al., the combined application of biochar and compost can alleviate the high mineralization rate of soil organic matter, phosphorus deficiency, and aluminum toxicity. By fixing carbon and improving soil physicochemical properties, it reduces the threat of soil water shortage or high salinity, and shows positive performance in the remediation of arid and saline soils [67]. Due to its high porosity and good water holding capacity, biochar can effectively promote the adhesion and proliferation of microbial cells through the rich functional groups such as active hydroxyl, carboxyl and amino groups on the surface. At the same time, biochar can be used as a sustainable substitute for peat and other non–renewable substrates, which can significantly enhance the persistence, viability and colonization effect of inoculated microorganisms in soil and plant roots, and then play an important role in soil biochemical processes, nutrient and carbon cycling, and pollution remediation [68]. In addition, biochar based probiotics can not only effectively promote plant growth, but also be used to repair soil contaminated by organic pollutants. Figure 6 illustrates applications of biochar in the remediation of organically contaminated soils.

The effects of biochar on soil remediation are not uniformly beneficial and may involve important trade offs. While biochar can enhance microbial activity and contaminant removal under appropriate conditions, certain biochars, particularly those produced at low pyrolysis temperatures or applied at high dosages, may release inhibitory compounds or alter nutrient availability, leading to microbial suppression. In addition, excessive adsorption or sequestration of organic pollutants can reduce their bioavailability to degrading microorganisms, thereby suppressing biodegradation despite apparent reductions in pollutant mobility. These effects are often dose dependent, as moderate biochar additions may improve soil structure and microbial functioning, whereas excessive application can disrupt soil ecological balance. Recognizing and addressing these contradictory responses is essential for optimizing biochar application strategies and ensuring both effective remediation and long term soil ecosystem health.

From a long term perspective, it is essential to clearly distinguish between pollutant immobilization and true remediation or mineralization when evaluating biochar based soil remediation. Pollutant immobilization primarily involves adsorption or sequestration processes that reduce contaminant mobility and bioavailability, thereby lowering short term ecological risk but not eliminating the contaminant mass. In contrast, true remediation or mineralization refers to the chemical or biological transformation of pollutants into less toxic or inorganic end products, which provides more durable benefits for soil functionality. Over extended time scales, aging of biochar and changes in soil conditions may weaken immobilization effects and lead to pollutant remobilization, whereas mineralization contributes to sustained risk reduction and ecosystem recovery. Therefore, distinguishing these processes is critical for accurately assessing long term remediation effectiveness and ecological safety.

For hydrophobic organic pollutants such as polycyclic aromatic hydrocarbons and petroleum hydrocarbons, biochar with high specific surface area and well–developed microporous structure usually exhibits excellent adsorption performance, and its removal efficiency increases with the degree of aromatization and the orderliness of carbon structure. High temperature pyrolysis biochar can achieve efficient fixation of such pollutants through hydrophobic distribution and π–π interactions, and has significant advantages in long–term stabilization and remediation. In contrast, pesticides, antibiotics, and some emerging organic pollutants usually have strong polarity or ionizable characteristics, and their removal performance depends more on the oxygen–containing functional groups, surface charges, and mineral phase composition on the surface of biochar. Biochar prepared at low temperatures or chemically modified often exhibits better adsorption and conversion capabilities in such polluted systems. In addition, the combined application of functionalized biochar with oxidants or microbial systems can further enhance the removal efficiency of recalcitrant organic pollutants. In addition to the nature of biochar, the interaction between biochar and soil may also be related to soil clay, soil organic matter content and pH value. According to Shang and Chi, two kinds of biochar extracted from wheat straw at 400 °C and 700 °C were used to study the changes in surface characteristics of biochar after aging in soil and its effect on phenanthrene adsorption. The effect of biochar on the adsorption of phenanthrene in soil is different due to different soils. The authors conclude that the surface polarity and micropore of biochar decrease the fastest in these mixtures, resulting in a significant reduction in hydrophobicity and pore filling [41]. Due to the soluble organic matter carried by biochar itself, which can serve as a carbon source to enhance the metabolic activity of microorganisms, a biochar microbe plant collaborative remediation framework has also been proposed, and the interaction and integration methods between biochar and bacteria, as well as their remediation efficiency and environmental friendliness, have been explored [69]. However, the limitations of a single remediation method include the sensitivity of plants to adverse soil conditions and the desorption of pollutants by biochar. In [5], Fang et al. proposed a combination of biochar and plant remediation for the remediation of organic polluted soil, involving the ability of biochar to enhance plant resilience, absorb pollutants, and degrade rhizosphere microorganisms. Meanwhile, plants can completely degrade pollutants adsorbed by biochar or pollutants in soil directly or indirectly through root exudates.

Biochar modification technology significantly compensates for the shortcomings of original biochar in adsorption selectivity, reaction driving force, and environmental adaptability by synergistically regulating pore structure, surface functional group composition, and reaction activity. Physical modification focuses on improving adsorption capacity and mass transfer efficiency, chemical modification enhances the molecular interaction between pollutants and biochar, mineral loading modification introduces catalytic and redox functions, while composite and functional modification achieve deep coupling of adsorption, conversion, and biodegradation.

Table 2 presents common biochar modification methods and their effects on the remediation performance of organic pollutants. The use of biochar for nitrogen retention in soil has a positive effect on reducing nitrogen loss and maintaining soil fertility. Shi et al. utilized a mixture of granular biochar and mineral urea to maintain nitrogen through strong interactions with surface proteins. They stated that the achieved effect may be mainly related to the retention of nitrogen bound to the surface of biochar/minerals, as well as the carbon bonds of biochar [70]. Cheng et al. used the immobilized microbial technology of biochar microbial synergy to repair PAHs–heavy metal complex pollutants. By analyzing the CO toxicity mechanism of the interaction of compound pollutants, which destroys the microbial membrane and metabolic pathway, the protective effects of biochar adsorption, reducing oxidative stress and promoting microbial colonization were evaluated [39].

From an integrated perspective, remediation performance is governed by the combined effects of biochar properties and soil conditions rather than by biochar characteristics alone. Biochars with high aromaticity, well developed pore structures, and abundant surface functional groups favor the adsorption and immobilization of hydrophobic organic pollutants, particularly in soils with neutral pH and moderate organic matter content. In contrast, in acidic or degraded soils, biochars with higher ash content and alkalinity are more effective by adjusting soil pH and stimulating microbial activity, thereby enhancing coupled adsorption and biodegradation processes. These cross study patterns underscore the importance of matching biochar physicochemical traits with site specific soil conditions and contaminant characteristics to achieve optimal remediation outcomes.

In Table 3, it presents the adsorption and behavior of biochar from different sources on various organic pollutants, including insecticides, polycyclic aromatic hydrocarbons, polychlorinated compounds, and herbicides.

Overall, biochar has emerged as a promising and sustainable soil amendment by simultaneously immobilizing organic pollutants, improving soil physicochemical properties, and stimulating microbial activity. Its remediation performance is governed by the intrinsic characteristics of biochar, soil conditions, and application strategies, highlighting the importance of tailoring biochar design to site–specific requirements. Despite its considerable potential, further mechanistic insights and long–term field–scale evaluations are required to ensure the effectiveness, stability, and environmental safety of biochar–based remediation approaches.

## 7. Discussion on Priorities and Trends

The application of biochar for the remediation of organically contaminated soils continues to face several challenges and uncertainties. The lack of unified standards for biochar production, coupled with variations in feedstock sources and preparation processes, results in substantial heterogeneity in physicochemical properties and highly site–specific and pollutant–specific remediation performance, thereby limiting the development of standardized and reproducible application strategies [62,95,96]. Moreover, most existing studies are restricted to laboratory or pot–scale experiments, with insufficient long–term field validation, leaving the aging behavior, adsorption stability, and sustained remediation effectiveness of biochar under realistic soil conditions inadequately understood.

In addition, the strong adsorption capacity of biochar may alter pollutant migration pathways, potentially increasing the risk of transport to deeper soil layers or groundwater. The mobility of biochar particles themselves may further facilitate pollutant migration through carrier effects, indicating that pollutant immobilization does not necessarily equate to permanent removal. Uncertainties also remain regarding the impacts of biochar amendments on soil microbial communities and ecosystem functions. Given the inherent complexity of soil systems, characterized by heterogeneity, complex microbial structures, and the coexistence of multiple pollutants, these challenges are further amplified. Although modified biochars often exhibit enhanced remediation efficiency, they may introduce higher costs and potential environmental risks. Therefore, future research should focus on strengthening mechanistic understanding, conducting long–term risk assessments, and establishing practical guidelines to support the safe and effective large–scale application of biochar for soil remediation [97,98,99]. The adsorption, catalytic, and biochar–microbe interaction mechanisms summarized in previous contents provide the mechanistic basis for rational biochar design, while the environmental risks and scalability challenges identified in last section highlight the need for long–term assessment and field–scale validation. By explicitly linking future directions with established mechanisms and unresolved constraints, this section aims to provide a coherent roadmap for advancing biochar–based soil remediation from laboratory research to practical applications. Based on the above analysis, this review identifies the following key directions for future research.

### 7.1. Rational Design and Engineering of Biochar Materials

A key priority in biochar–based soil remediation is the rational design of biochar with tailored physicochemical properties to target specific organic pollutants. Recent trends have shifted from conventional feedstock– and temperature–based optimization toward engineered biochars with controlled surface functionalization, pore structure, and mineral composition. Although modified and composite biochars often exhibit enhanced adsorption performance, their economic feasibility, environmental compatibility, and scalability require systematic assessment before large–scale application. A representative case study demonstrates that engineered biochar produced through sequential conversion and targeted surface modification can exhibit markedly enhanced adsorption performance and reusability. By combining hydrothermal carbonization of corn cob with subsequent Mg doping and pyrolysis, the resulting hydro–pyrochar showed a substantially modified surface chemistry and pore structure that favored strong and rapid Pb binding [100]. This rational design strategy increased the adsorption capacity by more than an order of magnitude compared with raw biomass and enabled stable reuse over multiple adsorption–desorption cycles with minimal performance loss. Such results highlight how stepwise conversion routes and purposeful chemical modification can effectively tailor biochar properties to achieve high adsorption efficiency, pollutant selectivity, and long term regeneration potential.

### 7.2. Mechanistic Understanding of Coupled Adsorption—Biodegradation Processes

Emerging evidence indicates that biochar–mediated remediation is governed by the synergistic interaction of adsorption, immobilization, and microbial degradation processes. Biochar not only serves as an adsorbent but also modifies microbial habitats and metabolic pathways, thereby influencing pollutant transformation. Future studies should integrate advanced material characterization, microbial ecology, and molecular–level analyses to elucidate the driving mechanisms of biochar–microbe–pollutant interactions under realistic soil conditions.

### 7.3. Translation from Laboratory Studies to Field–Scale Applications

Despite extensive laboratory and pot experiments, field–scale validation of biochar–based remediation remains limited. Soil heterogeneity, climatic variability, and long–term aging effects often lead to discrepancies between laboratory results and field performance. Developing standardized experimental protocols and long–term field monitoring frameworks is therefore essential to evaluate the persistence, stability, and practical effectiveness of biochar amendments in contaminated soils.

### 7.4. Environmental Risks and Sustainability Considerations

Assessing the environmental risks associated with biochar application has become an increasingly important research direction. Potential issues include changes in adsorption performance during aging, the release of biochar–associated contaminants, and unintended impacts on soil ecosystems. Incorporating life cycle assessment and risk–based evaluation approaches will be critical for balancing remediation efficiency with environmental safety and long–term sustainability. Environmental risks associated with biochar application can be broadly classified into short–term and long–term effects. Short–term risks mainly involve transient pH changes, release of labile components, and mobilization of inherent contaminants, particularly for low–temperature or high–ash biochars. Long–term risks are primarily related to biochar aging and surface oxidation, which may alter adsorption behavior and lead to pollutant remobilization. Such negative effects are more likely under specific soil–biochar combinations, highlighting the importance of site–specific biochar selection and long–term field assessment.

### 7.5. Integration with Complementary Remediation Technologies

An emerging trend in soil remediation research is the integration of biochar with other remediation strategies, such as phytoremediation, microbial augmentation, and chemical oxidation or reduction processes. These hybrid approaches aim to overcome the limitations of single techniques and enhance overall remediation efficiency. Future applications are likely to emphasize system–level, site–specific solutions that combine biochar with complementary technologies.

### 7.6. Molecular Descriptors and Structure–Activity Relationships Governing Biochar Performance

From the perspective of molecular descriptors and structure–activity relationships governing biochar performance, future research should move beyond empirical correlations and focus on establishing robust quantitative links between biochar molecular features and remediation outcomes. The systematic integration of molecular descriptors such as surface functional group density, degree of aromatic condensation, electron distribution, and redox active sites with pollutant specific properties will enable more reliable prediction of biochar performance. In addition, advanced spectroscopic and molecular scale characterization techniques, together with modeling approaches, can further elucidate how these structure–activity relationships regulate adsorption, catalytic transformation, and biochar microbe interactions. Such efforts are essential for the rational design of biochars with tailored functions and for enhancing the reliability and scalability of biochar based soil remediation strategies.

## 8. Conclusions

Biochar has become a promising multifunctional amendment for the remediation of organically contaminated soils owing to its tunable physicochemical properties, strong adsorption capacity, and beneficial interactions with soil microorganisms. This review consolidates recent advances in biochar–based remediation, emphasizing the key mechanisms and engineering strategies that underpin remediation performance and identifying critical challenges for scaling laboratory findings to field applications. Current evidence suggests that remediation efficiency is governed by synergistic processes, including hydrophobic partitioning, π–π electron donor–acceptor interactions, interfacial chemical interactions, and pore–filling effects, which collectively reduce pollutant mobility and bioavailability. In parallel, biochar can improve soil structure, regulate pH, enhance nutrient retention, and stimulate microbial activity, thereby facilitating coupled adsorption–biodegradation processes and enhancing long–term remediation outcomes. However, biochar performance remains highly dependent on feedstock selection, production conditions, pollutant characteristics, soil properties, and application strategies.

Future research should focus on the rational design of function–specific biochar, a deeper mechanistic understanding of biochar–pollutant–microbe interactions, and robust long–term field validation, alongside comprehensive risk assessments to ensure environmental safety and sustainability.

## Figures and Tables

**Table 2:** Common biochar modification methods.

Modification Category	References	Representative Pollutants	Main Property Enhancement	Remediation Effects	Dominant Interaction Mechanisms	Soil Conditions
Physical modification	[71,72]	PAHs, petroleum hydrocarbons	Increased surface area and porosity	Enhanced hydrophobic adsorption; reduced bioavailability	Enhanced adsorption via increased surface area and pore accessibility; physical entrapment	Coarse–textured or low organic matter soils; high contaminant mobility
Chemical modification	[73,74]	Antibiotics, pesticides	Enriched surface functional groups	Improved selective adsorption via electrostatic and π–π interactions	Surface complexation, electrostatic attraction, π–π interactions, selective adsorption	Neutral to alkaline soils; soils contaminated with aromatic or polar organic pollutants
Mineral loading modification	[70,75]	PAHs, POPs	Introduced catalytic and redox activity	Accelerated transformation of recalcitrant pollutants	Redox mediation, catalytic degradation, precipitation, co–immobilization	Redox–fluctuating or anaerobic soils; metal–associated or redox–sensitive pollutants
Composite and functionalized modification	[39,76]	Mixed organic pollutants	Multifunctional coupling effects	Synergistic adsorption–degradation and improved stability	Synergistic adsorption–biodegradation; microbial facilitation; stability enhancement	Biologically active soils; complex contaminant mixtures; long–term remediation scenarios

**Table 3:** Application of biochar in the remediation of organic pollutants in soil.

Organic Pollutant	References	Biochar Feedstocks	Influence
PAHs	[13,43,77,78]	Pine needles/hardwood/willow/sewage sludge	High–temperature biochar enhanced PAHs sorption via partitioning, reduced bioaccessibility, bioaccumulation and soil toxicity, and promoted microbial degradation
Phenanthrene	[79,80,81]	Pinewood/stinging nettle	Limited phenanthrene sorption on wood biochar, more pronounced in low–OC soils, with biochar– and soil OC–dependent sorption enhancement and up to 44% increased degradation
Polychlorinated Biphenyls	[82]	Soft wood	Resilient sorption dominated by hydrophobic partitioning and pore–filling
Pentachlorophenol	[83]	Bamboo	Reduced pentachlorophenol leaching via partitioning–driven biochar sorption.
Polychlorinated dibenzo–p–dioxins	[84]	Maize stover	Biochar significantly reduced particulate organic matter–extractable and bioavailable polychlorinated dibenzo–p–dioxins in soil by effective sorption and immobilization
Petroleum	[85]	Rice straw	Soil microbial degradation of petro–hydrocarbon enhanced by 20%
Ethinylestradiol	[86]	Sugarcane residue	Enhanced steroid sorption with desorption retardation, accompanied by reduced microbial mineralization
Tylosin	[87]	Hardwood	Stronger electrostatic interactions and surface complexation under alkaline conditions
Metalaxyl and Tebuconazole	[88]	Olive residues	Biochar decreased degradation and leaching of fungicides in soil
Diethyl phthalate	[89]	Bamboo	90% sorption effect
Herbicides	[90]	Poultry waste	Poultry biochar showed great sorption capacity for norflurazon and fluridone
Diuron	[40]	Eucalyptus	Progressive sorption equilibrium and increased availability of active sorption sites
Carbofuran and chlorpyrifos	[90]	Eucalyptus	Stronger pesticide adsorption with increasing pyrolysis temperature and biochar application rate
Acetochlor and Atrazine	[91,92]	Woodchip/green waste	Enhanced sorption via hydrophobic interactions and pore–filling effects
Norflurazon and fluridone	[13,93]	Wheat straw/swine manure	Hydrophobic partitioning and π–π interactions with aromatic biochar surfaces
Simazine	[94]	Hardwood	Simazine biodegradation inhibited and leaching decreased

## Data Availability

No new data were created in this review.
